# Enrichment of anchoring sites by introducing supramolecular halogen bonds for the efficient perovskite nanocrystal LEDs

**DOI:** 10.1038/s41377-023-01266-4

**Published:** 2023-09-04

**Authors:** Po Lu, Ting Li, Min Lu, Cheng Ruan, Siqi Sun, Zhennan Wu, Yuan Zhong, Fujun Zhang, Yanbo Gao, Yaowei Huang, Yang Wang, Junhua Hu, Fengping Yan, Yu Zhang

**Affiliations:** 1 State Key Laboratory of Integrated Optoelectronics and College of Electronic Science and Engineering, Jilin University, Changchun, China; 2 School of Electronic and Information Engineering, Beijing Jiaotong University, Beijing, China; 3 Changchun Cedar Electronics Technology Co., Ltd., Changchun, China; 4 Changchun Institute of Optics, Fine Mechanics and Physics, Chinese Academy of Sciences, Changchun, China; 5 Key Laboratory of Materials Physics of Ministry of Education Department of Physics and Engineering, Zhengzhou University, Zhengzhou, China

**Keywords:** Lasers, LEDs and light sources, Inorganic LEDs

## Abstract

Considering the multi-functionalization of ligands, it is crucial for ligand molecular design to reveal the landscape of anchoring sites. Here, a typical triphenylphosphine (TPP) ligand was employed to explore its effect on the surface of CsPbI_3_ perovskite nanocrystals (PNCs). Except for the conventionally considered P-Pb coordination, an P-I supramolecular halogen bonding was also found on the NC surface. The coexistence of the above two types of bonding significantly increased the formation energy of iodine vacancy defects and improved the photoluminescence quantum yield of PNCs up to 93%. Meanwhile, the direct interaction of P and I enhanced the stability of the Pb-I octahedra and dramatically inhibited the migration of I ions. Furthermore, the introduction of additional benzene rings (2-(Diphenylphosphino)-biphenyl (DPB)) increased the delocalized properties of the PNC surface and significantly improved the charge transport of the PNCs. As a result, the DPB passivated CsPbI_3_ NCs based top-emitting LEDs exhibite a peak external quantum efficiency (EQE) of 22.8%, a maximum luminance of 15, 204 cd m^−2^, and an extremely low-efficiency roll-off of 2.6% at the current density of 500 mA cm^−2^.

## Abstract (web summary)

Optoelectronic performances of perovskite nanocrystals were improved by the introduction of new anchoring points and delocalized benzene rings. Corresponding LEDs achieved high EQE of 22.8% and low efficiency roll-off.

## Graphical abstract

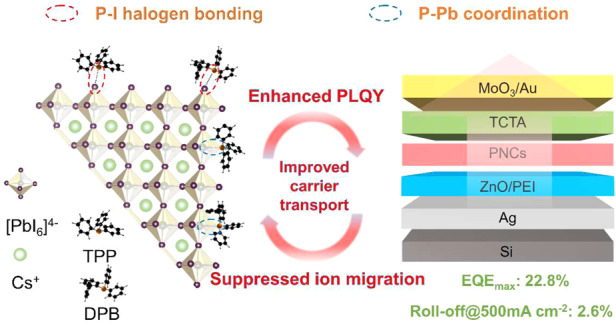

## Introduction

Colloidal semiconductor nanoparticles can be viewed as a complex of an inorganic single crystal core and a monolayer of organic ligands. The location and type of ligand anchoring on the nanocrystal surface are critical to the nanocrystal morphology, size, bonding patterns, adsorption-desorption processes, and overall stability, optoelectronic properties, etc.^1,2^. In additon, it is also the key consideration in the design of ligand molecule, ideal nanocrystal materials and their applications. Especially in the perovskite nanocrystals (PNCs) with the nature of soft lattices, the bonding environment of ligand has played a paramount role in determining the optoelectronic properties and stability of PNCs^3–8^. However, there is a great challenge to decipher the molecular images of dynamic organic-inorganic interfaces. And the idealization, precision, and flexibility of designing ligand molecules are desperately needed to guide the performance and field development of PNC materials.

The current perception of anchoring sites is in the form of “point-to-point”, e.g., the L-type ligands (alkylamine, alkylphosphine oxide, etc.) have been proved to anchor the unsaturated Pb^2+^ sites or Cs^+^ sites to improve the optical properties and stability of PNCs^9,10^. Alkylammonium salts of X-type ligands and Z-type ligands (sodium salts, potassium salts, etc.) are also chosen to anchor halogen anion to inhibit ion migration^11,12^. Obviously, one effective way to passivate the defect sites on the surface of PNCs is to select different ligand molecules or even introduce multifunctional groups on a single ligand, thereby creating multiple anchoring sites^4,13,14^. However, the interaction between functional groups and anchoring sites as well as the synergistic and repulsive properties between functional groups are not yet fully understood, which hinders the idealized design of high-performance PNC materials and devices.

Here, we revealed a new anchoring site on the surface of CsPbI_3_ PNCs by employing the classical triphenylphosphine (TPP) ligand. It is found that, in addition to the conventionally considered P-Pb coordination interaction^15–17^, P and I can also form an unexpected halogen bonding interaction. The appearance of new anchoring site can effectively passivate the PNC’s surface defects and suppress ion migration, thus resulting in significant improvement in optical properties and stability of PNCs. Furthermore, the 2-(Diphenylphosphino)-biphenyl (DPB) containing an additional benzene ring compared to TPP was used as ligands to enhance the electrical properties of PNCs. The extra benzene ring not only improved the interactions between P and the PNC surface, but also enhanced electron delocalization, leading to improved carrier injection and transport. Ultimately, we prepared bottom-emitting and top-emitting LED devices, respectively. The maximum EQEs of the devices are 19.2% (TPP, bottom-emitting), 21.4% (TPP, top-emitting), 21.6% (DPB, bottom-emitting) and 22.8% (DPB, top-emitting), which are more than twice the EQEs of the pristine LEDs.

## Results

**Fig. 1:**
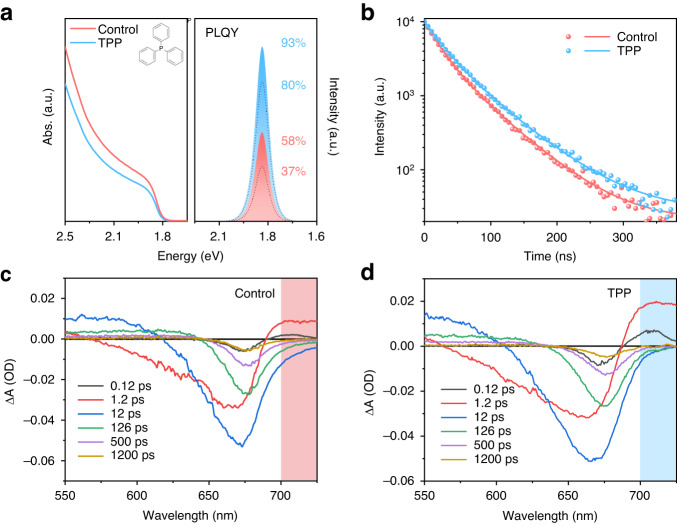
The optical properties of different PNCs. **a** UV−vis absorption (left) and PL spectra (right) (The inset is the molecular structural formula of TPP), **b** time-resolved normalized PL decay spectra for the PNCs with and without TPP passivation. (The filled areas are the PLQYs of the PNC solution, and the dot lines are the PLQYs of the corresponding films.) TA spectra of **c** control PNC film and **d** TPP passivated PNC films. The square-shaded areas highlight the band tail absorption

The PNCs were synthesized by the conventional hot-injection method (details in **Materials and methods**). Figure 1a shows the UV−vis absorption and photoluminescence (PL) spectra of the as-synthesized PNCs with and without TPP passivation. After TPP passivation, the absorption and PL peaks of PNCs didn’t change, but the photoluminescence quantum yields (PLQYs) of PNC solutions improved from 58% to 93% (Table S1). The transmission electron microscope (TEM) images and X-ray diffraction (XRD) patterns (Fig. S1) confirm that both samples are pure cubic phase with the same interplanar spacing and similar diffraction patterns. The corresponding time-resolved PL decays are shown in Fig. 1b. Both curves were fitted by biexponential decay function of ${A}_{1}{e}^{-t/{\tau }_{1}}+{A}_{2}{e}^{-t/{\tau }_{2}}$ and the average lifetimes are 42 (control) and 50 ns (TPP), respectively, which means the effective defect passivation from TPP^18–23^.

Transient absorption (TA) spectroscopy has been implemented to gain more insights into the carrier dynamics for the CsPbI_3_ PNC solution. As shown in Fig. S2 and S3, the two negative photoinduced bleach (PB) profiles, labeled respectively as PB1 and PB2, can be attributed to the ground-state bleach and the hot-exciton induced bleach, respectively. The other two positive photoinduced absorption (PA) profiles, labeled respectively as PA1 and PA2, are arising from the lowest excitonic state and hot charge carriers (hot-exciton or higher-lying excitonic states)^24,25^. The corresponding global analysis is performed to retrieve the decay-associated spectra (DAS), and the respective time constants are given in Fig. S3. The τ_1_, τ_2_, τ_3_ and τ_4_ components refer to the process of intraband hot-exciton relaxation, exciton relaxation to the lowest excited state, exciton trapping to the trap states and exciton recombination, respectively^26,27^. The decrease of τ_2_ and τ_3_ means the promoted coupling between the higher exciton state and the lowest exciton state and the coupling between the lowest exciton state and defect state for the TPP passivated PNCs, which is beneficial to the luminescence enhancement^24^. Considering that the ligand passivation can also greatly improve the PLQY of the PNC film, we proceed TA measurements to explore the carrier state of the PNC films. Figure 1c, d and Fig. S4 show the TA spectra of different PNC films recorded at different delay times. It can be seen that the PB1 signal of the control film shows distinct bands (>700 nm) in different delay time scales, which can be attributed to the sub-bandgap trap states (e.g., V_I_) caused by the shedding of ligands and the aggregation of PNCs after film formation^28,29^. The tail absorption in the TPP passivated film is significantly weakened, indicating that the ligand passivation has an excellent protective effect on the surface of PNCs.

**Fig. 2:**
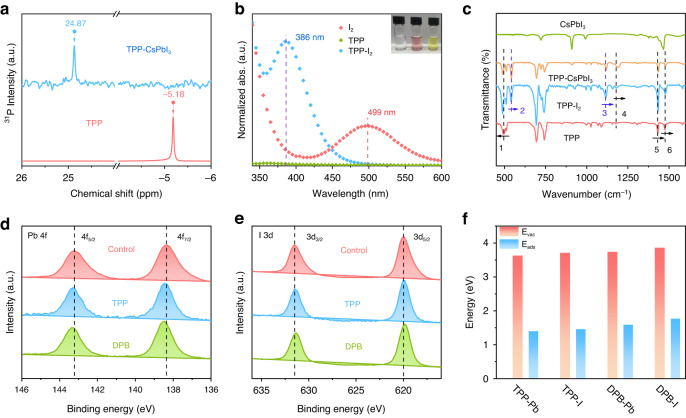
The interaction of different ligands with the PNC surface. **a** ^31^P NMR spectra for TPP and TPP-CsPbI_3_ NCs. **b** UV–vis absorption spectra for I_2_, TPP, and TPP-I_2_. The inset is the photos of toluene solution including TPP (left), I_2_ (mid) and TPP-I_2_ (right). **c** FTIR spectra for TPP, TPP-I_2_, TPP-CsPbI_3_ NCs and CsPbI_3_ NCs. XPS spectra of **d** Pb 4 f and **e** I 3d of NCs passivated by different ligands. **f** Absolute value of the estimated adsorption energy (E_ads_) of different ligands on the CsPbI_3_ PNC surface, and the corresponding formation energy of surface V_I_ (E_vac_)

To explore the location of anchoring sites of TPP on the PNC surface, we first performed ^31^P nuclear magnetic resonance (NMR) spectroscopy (Fig. 2a). There is a chemical shift in TPP-CsPbI_3_ compared to TPP, indicating that the P-containing functional groups in TPP interact with the surface of CsPbI_3_ PNCs, resulting in a change in the coordination environment of P^30^. To further understand the interaction, we conducted different analogy experiments. Considering the role of the halogen bond between the P-containing molecule and the I-containing molecule^31^, we explored the interaction possibility between TPP and iodine (I_2_). Figure 2b shows the UV-vis absorption spectra of I_2_, TPP and TPP-I_2_ mixture. After the addition of TPP, the absorption peak of I_2_ at 499 nm shifted to 386 nm, demonstrating a strong interaction between P and I_2_ and the formation of the halogen bonds^31^. This interaction is more visually illustrated by the change in solution color, where the purple-red solution of I_2_ turned yellow after the addition of TPP.

FTIR measurements given in Fig. 2c were used to further clarify the chemical interaction between ligand additives and CsPbI_3_ PNCs. The variations of vibration peaks in TPP are complicated. For convenience, we have labeled the shifted peaks with different colors in Fig. 2c. The characteristic peak 1 of TPP shifted to the lower wavenumber, while the vibrational peaks 4, 5 and 6 shifted to the opposite direction. Morover, new vibration peaks 2 (539 cm^−1^) and 3 (1115 cm^−1^) appeared in the TPP-I_2_, which may be attributed to the interaction between TPP and I_2_. Because of the special electron cloud distribution of I_2_, the electrophilic region on the electrostatic potential surface of I acts as a halogen bond donor, and the nucleophilic P belonging to the halogen bond acceptor promotes the formation of I^…^P halogen bond^31,32^. In addition, the FTIR spectrum of TPP-passivated PNCs also show two additional peaks 2 and 3, but they shift to 542 cm^−1^ and 1120 cm^−1^, respectively. This suggests that the I^…^P supramolecular interaction in TPP passivated CsPbI_3_ PNCs is similar but not identical to that of TPP-I_2_, which is attributed to the different chemical environment of I atoms in I_2_ and CsPbI_3_. To further verify the role of P-containing functional groups, we introduced (Diphenylphosphino)-biphenyl (DPB) to passivate CsPbI_3_ PNCs. As shown in Fig. S5, the DPB passivated PNCs exhibit higher PLQYs of 95% (solution) and 85% (film) and prolonged PL lifetime of 52 ns than TPP passivated PNCs. Furthermore, the changes in TA, ^31^P NMR, UV-vis, and FTIR spectra are consistent with those observed in TPP passivated PNCs (Fig. S6). These findings suggest that the interaction between DPB and PNCs is similar to that of TPP, but the passivation effect is stronger.

XPS measurements also confirm the different chemical states of the different PNC films, as shown in Fig. 2d, e. The Pb 4 f and I 3d spectra in control film exhibit two contributions, 4f_7/2_ and 4f_5/2_, located at 138.3 and 143.2 eV, 3d_3/2_ and 3d_5/2_, located at 631.1 and 619.6 eV, respectively. The Pb 4 f spectra of TPP and DPB passivated PNC films shift to the higher binding energy due to the strong binding between the Pb and P functional groups^33,34^. The I 3d spectra of TPP and DPB passivated PNC films shift to the lower binding energy, which can be considered as the result of the interaction of the nucleophilic atom P in TPP or DPP with the I in PNCs to give electrons to the electrophilic region of I^31,35^. The high-resolution XPS spectra of N, P, O, and atomic ratio data are shown in Fig. S7. The peaks of N and O don’t change significantly, and the peak of P appears after TPP and DPB treatment, indicating that the two ligands exist on the surface of PNCs. In addition, the proportion of N and O decreased after ligand treatment, indicating that a portion of oleylamine and oleic acid are replaced by TPP and DPB. To further illustrate the nature of different ligands bonding to the PNC surface, the DFT calculations were performed with the results shown in Fig. 2f and Fig. S8. The adsorption models of guest molecules and CsPbI_3_ are designed. DFT is used to calculate the adsorption energy of ligands (E_ads_) and the corresponding formation energy of surface I vacancies (E_vac_). The results show that the adsorption interface of guest molecules can fully combine with CsPbI_3_ to form a stable structure, which has a certain effect on the crystal structure. Through sufficient structural relaxation, a stable structural model was obtained, and the E_ads_ values of different ligands with Pb or (and) I on the surface of PNCs were estimated. Intriguingly, DPB shows larger E_ads_ values with both Pb and I in comparison to TPP, suggesting that it is possible to stabilize the surface of the PNCs by reducing surface distortion^17^. Furthermore, the E_ads_ values of P with I are higher than that with Pb, indicating that TPP and DPB preferentially interact with I on the PNC surface rather than Pb. This means that I ions can be well remained on the PNC surface, thus leading to increased E_vac_ of V_I_ and enhanced phase stability (Fig. S9).

**Fig. 3:**
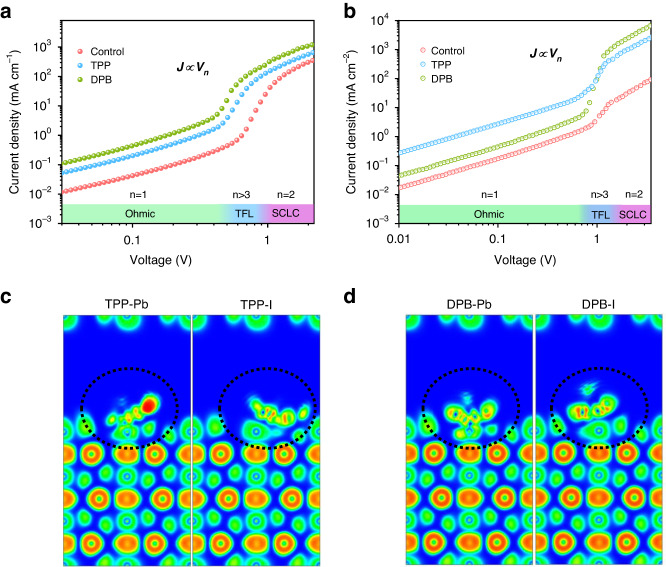
The electronic properties of different PNCs. Current densities as a function of bias voltage for **a** electron-only devices and **b** hole-only devices. The electron-only device structure is ITO/ZnO/PEI/PNCs/Ag; the hole-only device structure is ITO treated with 1,2-dichlorobenzene/PNCs/TCTA/MoO_3_/Au. The electron localization function (ELF) of **c** TPP and **d** DPB ligands on the CsPbI_3_ surfaces (red is high value region, green is low value region)

To demonstrate the impact of different ligands on the electrical properties of thin films, we utilized the space-charge-limited current (SCLC) method (as detailed in **Materials and Methods**) to determine the carrier mobilities of various samples. The results are visualized in Fig. 3a, b, and the corresponding calculations are summarized in Table S2. The electron and hole mobilities are 1.3 × 10^−3^ and 3. 0× 10^−4^ cm^2^ V^−1^ s^−1^ for Control sample, 2.0 × 10^−3^ and 7.8 × 10^−4^ cm^2^ V^−1^ s^−1^ for TPP passivated PNCs, 2.8 × 10^−3^ and 9.2 × 10^−4^ cm^2 ^V^−1^ s^−1^ for DPB passivated PNCs, respectively. The improvements of carrier mobilities are mainly benefited from the reduced carrier scattering from the defects, the abbreviated ligand length and the delocalization of the benzene ring π electrons by the ligand passivation, which lowered the energy barrier of charge transfer between PNCs^36,37^. The electron localization function (ELF) was calculated to compare the delocalization of different ligands on the PNC surface, as shown in Fig. 3c, d. In the region surrounded by the equivalent bread of ELF with high value (red region), the electrons are highly localized and are not easy to run out. On the contrary, in those regions with low ELF values (green region), the electron localization is weak and the electrons can easily move freely in such a region or free to leave the domain. Obviously, the DPB ligand position shows stronger delocalization performance, which is beneficial to charge carrier transport^38^.

**Fig. 4:**
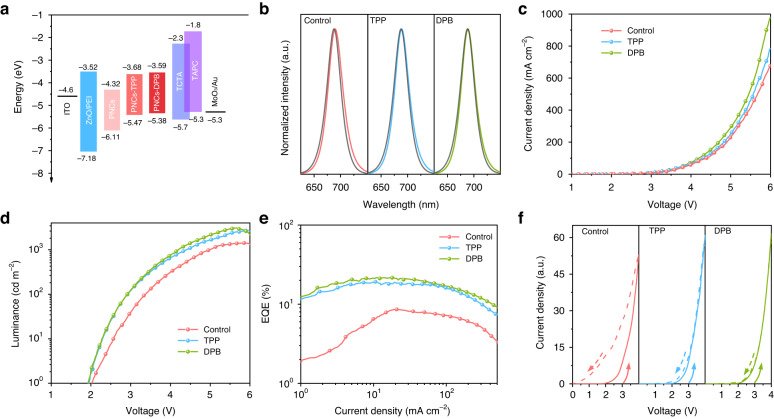
The device performances of bottom-emitting LEDs. **a** Device energy-level diagram for each functional layer in the LEDs. **b** Spectral comparison of PL of different films and EL of PNC LEDs. **c** current density versus bias voltage, **d** Luminance versus bias voltage, **e** EQE versus current density curves of LEDs based on different ligands passivated PNCs. **f** Current density versus bias voltage curves of these devices under forward and reverse voltage scans (the arrows indicate the direction of the scanning voltage)

The pristine and passivated CsPbI_3_ PNCs were used as emitting layers to fabricate electroluminescence (EL) LEDs of different device strcutures (bottom-emitting and top-emitting). A schematic diagram of the bottom-emitting perovskite LED with a multilayered structure of ITO/ZnO/Polyethylenimine (PEI)/PNCs/4,4′,4″-tris(carbazol-9-yl) triphenylamine (TCTA)/ 1,1-bis-(4-bis(4-tolyl)-aminophenyl) cyclohexene (TAPC)/MoO_3_/Au is given in Fig. S10a. The energy levels of different PNC films were explored by UPS measurement (Fig. S10b, c) and the Tauc plots (Fig. S10d), and the corresponding energy band diagram for all functional layers is shown in Fig. 4a. The passivated PNCs based devices exhibit more suitable energy level structures compared with the control device, which can promote the radiative recombination of carriers in emitting layers (Fig. S11, Table S3). The EL characteristics of different PNCs based devices are shown in Fig. 4b–e and Table 1. The maximum luminance increases from 1419 cd m^−2^ (Control) to 2731 cd m^−2^ (TPP) and 3088 cd m^−2^ (DPB), which benefits from the improvement on PLQY and carrier mobilitiy after ligand passivatin. In additon, the turn-on voltages of the passivated PNCs based devices are reduced from 2.0 V to 1.9 V due to the reduced hole injection barrier. Consequently, the maximum EQEs of 19.2% and 21.6% are respectively achieved for TPP and DPB based devices, which is significantly higher than 8.5% EQE of control device. Furthermore, we tested the current-voltage curves of LEDs based on different ligands passivated PNCs under forward and reverse voltage scans to explore ion migration (Fig. 4f). The ligands passivated PNCs based devices clearly show smaller hysteresis than that of control device, demonstrating that ligand passivation can help to stabilize PNC surface and impede ion migration. Wondrously, DPB based device shows almost non-existent hysteresis, which is attributed to the stronger interaction between ligands and PNC surface, thus stabilized the crystal structure and suppressed ion migration well.

**Table 1:** Device performances of LEDs based on different ligands passivated PNCs and different device structure

Sample	EL peak (nm)	V_on_ (V)	Luminance_max_ (cd m^−2^)	EQE_max_ (%)
Control (B)	690	2.0	1419	8.5
Control (T)	689	2.4	4169	11.3
TPP (B)	689	1.9	2731	19.2
TPP (T)	688	2.2	13475	21.4
DPB (B)	689	1.9	3088	21.6
DPB (T)	688	2.2	15204	22.8

B Bottom-emitting, T Top-emitting

**Fig. 5:**
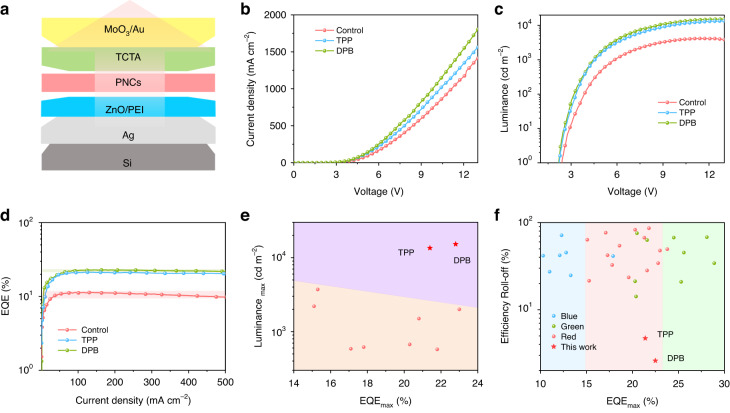
The device performances of top-emitting LEDs. **a** A schematic architecture of PNC based top-emitting LEDs. **b** Current density versus bias voltage, **c** luminance versus bias voltage, **d** EQE versus current density curves of LEDs based on different ligands passivated PNCs. **e** Maximum luminance (Luminance_max_) versus maximum EQE (EQE_max_) of red-emitting perovskite LEDs reported in the literature (Table S4). **f** Efficiency roll-off versus EQE_max_ at a given current density for blue-, green- and red-emitting perovskite LEDs reported in the literature (Table S4). The red star stands for the TPP and DPB based top-emitting LEDs in this work

Furthermore, the top-emitting CsPbI_3_ NC LEDs with a multilayered structure of Si/Ag/ZnO/PEI/PNCs/TCTA/MoO_3_/Au were fabricated (Fig. 5a). The performance characteristics of the top-emitting devices based on different ligands passivated PNCs are shown in Fig. 5b–d and Table 1. The top-emitting PNC LEDs exhibit significantly higher current density and luminance than the bottom-emitting devices, which is due to the increased light extraction efficiency caused by the strong microcavity resonance effect between the bottom and top electrodes^39^. A peak luminance of 15204 cd m^−2^ for DPB passivated PNC LED was achieved, which is the highest value reported for red perovskite LEDs (Fig. 5e and Table S4). The TPP and DPB passivated top-emitting CsPbI_3_ NC LEDs also have higher EQE than bottom-emitting devices, and the maximum EQEs are 21.4% and 22.8%, respectively. More importantly, the TPP and DPB passivated top-emitting CsPbI_3_ NC LEDs show much lower efficiency roll-off at high current density and luminance. At the current density of 500 mA cm^−2^, the pristine CsPbI_3_ NC LED has an efficiency roll-off of 13.2%, while the TPP and DPB passivated CsPbI_3_ NC LEDs have an efficiency roll-off of only 4.7% and 2.6%, respectively, which is the best performance among reported perovskite LEDs (Fig. 5f and Table S4). The decrease in efficiency roll-off is attributed to the suppression of emission quenching due to Joule heating and the suppression of Auger recombination due to unbalanced charge carrier injection^39–42^. The repeatability of top-emitting LED devices was also tested, as shown in Fig. S12. The average EQE_max_ of the Control, TPP, and DPB-based devices are 10.2%, 19.9 % and 21.2%, respectively. Fig. S13 shows the device operating stability. The TPP and DPB passivated top-emitting CsPbI_3_ NC LEDs exhibit a T_50_ lifetime of nearly 30 and 35 h, which is 30 and 35 times longer than the control top-emitting device, respectively.

## Discussion

In conclusion, we adopt tri-coordinated trivalent P organic molecules (TPP and DPB) to explore their anchoring sites with the surface of CsPbI_3_ PNCs. TPP and DPB can interact with both Pb and I on the PNC surface, which can significantly suppress the formation of V_I_ defects and stabilize the cubic symmetry of the PNCs by reducing surface distortion, thus leading to more superior luminescence performance and material stability. In addition, the short-chain ligands and delocalized nature of benzene rings enhanced the carrier transport ability, and a more optimized energy level structure helped more balanced carrier recombination. Finally, the BPB passivated PNC based top-emitting LEDs achieved a peak EQE of 22.8% and a extremely low efficiency roll-off of 2.6% at the current density of 500 mA cm^−2^. The selection of multifunctional anchoring sites provides a new strategy for improving the optoelectronic properties of PNCs and devices.

## Materials and methods

### Materials

CH_3_COOCs (CsAc, 99.9%), Oleic acid (OA, 90%), Oleylamine (OLA, 70%), octadecene (ODE, 90%), ethyl acetate (99.9%), triphenylphosphine (TPP, ≥95%) and 2-(Diphenylphosphino)-biphenyl (DPB, 98%) were purchased from Aladdin, and lead iodide (PbI_2_, 99.999%) were obtained from Sigma-Aldrich. Toluene (99.5%) was purchased from Beijing Chemical Factory. All chemicals were used directly without further purification.

### Synthesis of CsPbI_3_ NCs

For the typical synthesis of CsPbI_3_ NCs, 10.0 mL ODE, 0.173 g PbI_2_, 1 mL OA and 1 mL OLA were loaded into a 50 mL three-neck flask which were degassed and dried under vacuum for 45 min at 120 °C. Then the temperature was increased to 170 °C and 1.0 mL Cs-oleate solution (0.288 g CsAc, 1 mL OA and 14.0 mL ODE were loaded into a 20 mL vial which sealed and stirred at 120 °C until a clear solution was obtained) was quickly injected. Five seconds later, the reaction mixture was immediately cooled down to room temperature by an ice-water bath. The product was centrifuged at 5000 rpm for 10 min whose precipitate was reserved and dissolved in equal volumes of toluene and ethyl acetate to further purify by centrifuging at 10,000 rpm for 8 min. Eventually, the NCs were dispersed in toluene for use. For the synthesis of NCs passivated by different ligands, the 0.3 g TPP or 0.0203 g DPB were added to the PbI_2_ solution.

### Device fabrication

Bottom-emitting device structure: Indium tin oxide (ITO) glass substrates were cleaned by UV-ozone treatment for 10 min. The ETL was prepared via spin-coating ZnO NC solution onto the ITO substrates at 1000 rpm for 30 s and annealed in air at 150 °C for 10 min. Then, a solution of polyethyleneimine (PEI) dissolved in 2-methoxyethanol (0.2% mass ratio) was spin-coated onto the ZnO film at a speed of 3000 rpm for 50 s and annealed at 125 °C for 10 min in the glovebox. PNC emitting layer was then deposited by spin-coating at 2000 rpm for 50 s. TCTA, TAPC, MoO_3_ and Au were then sequentially deposited by thermal evaporation in a vacuum deposition chamber (1 × 10^−7^ Torr). (TCTA and TAPC were deposited by co-evaporation with a thermal evaporation rate of 0.5 Å s^−1^)

Top-emitting device structure: Silicon wafers were cleaned successively using soap, deionized water, ethanol, acetone, and isopropanol. A 150 nm Ag film was deposited onto the silicon substrate via thermal evaporation, and a solution of ZnO (50 mg mL^−1^) was spin-coated on top of the Ag film at 1000 rpm for 30 s and annealed in air at 150 °С for 10 min. The substrate was transferred into a N_2_ glove-box, and a solution of PEI dissolved in 2-methoxyethanol (0.2% mass ratio) was spin-coated onto the ZnO film at a speed of 3000 rpm for 50 s and annealed at 125 °C for 10 min in the glovebox. PNC emitting layer was then deposited by spin-coating at 1000 rpm for 50 s. TCTA, MoO_3_ and Au were then sequentially deposited by thermal evaporation in a vacuum deposition chamber (1 × 10^−7^ Torr).

### Characterizations

The UV–vis absorption spectra were measured with Shimadzu UV-2550 spectrophotometer. The PL spectra of the PNCs and the EL spectra of LEDs were obtained by the S3 Ocean Optics spectrometer. X-ray diffraction (XRD) patterns were acquired by using Bruker D8 Advance X diffractometer (Cu Kα, λ = 1.5406 Å). FTIR spectra were measured by using an IFS-66V/S FITR spectrophotometer. ^31^P NMR data were collected on a Bruker NMR spectrometer (AVANCE III, 600 MHz). Time-resolved PL measurements were performed with a time correlated single-photon counting system of the FLS920P Edinburgh spectrometer. The absolute PLQYs of the PNCs (in the form of solution and film) were measured on a fluorescence spectrometer (FLS920P, Edinburgh Instruments) equipped with an integrating sphere. TEM images were collected on a FEI Tecnai F20 microscope. The current-voltage characteristics of the devices were measured with a Keithley 2612B source meter and the LED luminance was determined using a Photo Research Spectra Scan spectrometer PR650, and the stability measurement for the device was carried out by using it under a nitrogen atmosphere in the glovebox at room temperature (20 ± 5 °C) without any encapsulation. The femtosecond transient absorption spectra were collected using a pump–probe configuration (pump wavelength: 400 nm, pump light intensity: 120 μJ/cm^2^). Ultraviolet photoelectron spectroscopy (UPS) with a multi-technique surface analysis system (VG Scienta R3000) with excitation energy of 21.218 eV was performed to measure the energy levels of perovskite NCs. The complex impedance measurements of the sensors were performed by using an impedance analyzer (Solartron 1260 and Solartron 1287) in the frequency range of 1–100,000 Hz. The amplitude of the AC potential signal was fixed at 110 mV and the applied bias voltage was set as 0.3 V.

### General information of DFT simulations

The structural optimizations and electronic structure calculations are performed based on DFT as implemented in the Vienna Ab Initio Simulation Package (VASP) code^43^ and the projector augmented wave (PAW) method with a cutoff energy of 600 eV^44^. All of configurations of CsPbI_3_ based models were fully optimized. The generalized gradient form (GGA) of the exchange-correlation functional (Perdew-Burke-Ernzerhof 96, PBE) was adopted^45,46^. A revised Perdew–Burke–Ernzerhof generalized gradient approximation (PBEsol)^47,48^ was used for the exchange-correlation. PBEsol functional has been introduced to improve the equilibrium properties of solid^49^. Valence-core interactions were described by projector-augmented-wave (PAW) pseudopotentials^50^. The Brillouin zone sampling is carried out using the (3 × 3 × 1) Monkhorst-Pack grids for surface and Gamma for the structure. The convergence tolerance of energy is 1 × 10^−5^ eV, maximum force is 0.002 eV Å^−1^, and maximum displacement is 0.002 Å^44^. The ultrasoft scalar-relativistic pseudopotentials were used to describe the electron-ion interactions by explicitly treating electrons for H (1s^1^), N, O and C (2s^2^, 2p^2^), I (5s^2^, 5p^2^), Cs (5s^2^, 5p^6^, 6s^1^), and Pb (5d^10^, 6s^2^, 6p^2^).

The vacancy formation energy (*E*_vac_) and adsorption energy (*E*_ads_) of defects was defined as:

1 $${E}_{{vac}}={E}_{{Total}}-{{E}}_{\left({{\rm{CsPbI}}}_{3}\right)}{\,+\,{E}}_{({\rm{I}})}$$

2 $${E}_{{ads}}={E}_{{Total}}-{E}_{\left({{\rm{CsPbI}}}_{3}\right)}-{E}_{({\rm{guest\; molecules}})}$$

${E}_{{Total}}$ is the energy of the relevant supercell of guest molecules adsorbed with CsPbI_3_, ${E}_{\left({{\rm{CsPbI}}}_{3}\right)}$ is the energy of the relevant supercell of CsPbI_3_, ${E}_{({\rm{guest\; molecules}})}$ is the energy of ${\rm{guest\; molecules}}$, ${E}_{\left({\rm{I}}\right)}$ is the energy of I atom.

### Calculation of carrier mobility

The electron and hole mobilities of different PNC films were estimated by fitting the space-charge-limited-current (SCLC) region with Mott-Gurney law J = 9εε_0_μV^2^/8L^3^, where ε and ε_0_ are the relative dielectric constant of the PNCs and the vacuum permittivity, respectively, J is the current density, μ is the carrier mobility, V is the applied voltage, and L is the thickness of the obtained PNC film^51^.

### Supplementary information

Revised Supporting Information - clear
