# Supplementary material for: Enrichment of anchoring sites by introducing supramolecular halogen bonds for the efficient perovskite nanocrystal LEDs
Source: Light Sci Appl. 2023 Sep 4;12:215. doi: 10.1038/s41377-023-01266-4 (PMC10477334; doi:10.1038/s41377-023-01266-4)
Supplement: Supplementary file 1 — Revised Supporting Information - clear [file 41377_2023_1266_MOESM1_ESM.docx]

**Supplementary Information**

**Enrichment of anchoring sites by introducing supramolecular halogen bonds for the efficient perovskite nanocrystal LEDs**

Po Lu^1^, Ting Li^2^, Min Lu^1^*, Cheng Ruan^3^, Siqi Sun^1^, Zhennan Wu^1^, Yuan Zhong^1^, Fujun Zhang^1^, Yanbo Gao^1^, Yaowei Huang^3^, Yang Wang^3, 4^*, Junhua Hu^5^, Fengping Yan^2^*, Yu Zhang^1^*

Correspondence: Min Lu ([lumin@jlu.edu.cn](mailto:lumin@jlu.edu.cn)), Yang Wang ([wangy@ccxida.com](mailto:wangy@ccxida.com)), Fengping Yan (fpyan@bjtu.edu.cn), Yu Zhang (yuzhang@jlu.edu.cn)

^1^State Key Laboratory of Integrated Optoelectronics and College of Electronic Science and Engineering, Jilin University, Changchun 130012, China

^2^School of Electronic and Information Engineering, Beijing Jiaotong University, Beijing 100044, China

^3^Changchun Cedar Electronics Technology Co., Ltd., Changchun 130103, China

^4^Changchun Institute of Optics, Fine Mechanics and Physics, Chinese Academy of Sciences, Changchun 130103, China

^5^Key Laboratory of Materials Physics of Ministry of Education Department of Physics and Engineering, Zhengzhou University, Zhengzhou, 450052, China


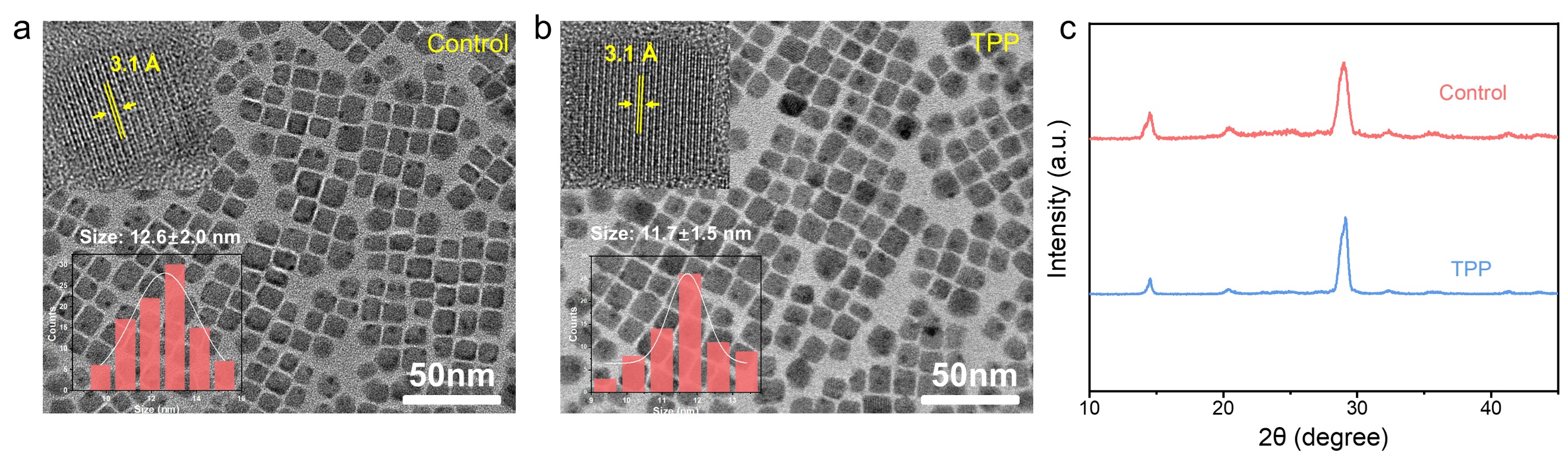


**Fig. S1.** TEM images of (a) pristine CsPbI_3_ NCs and (b) TPP passivated CsPbI_3_ NCs. The top left insets are their corresponding high-resolution TEM images and the bottom left insets shows their corresponding size distribution histograms. (c) XRD patterns of both NCs.


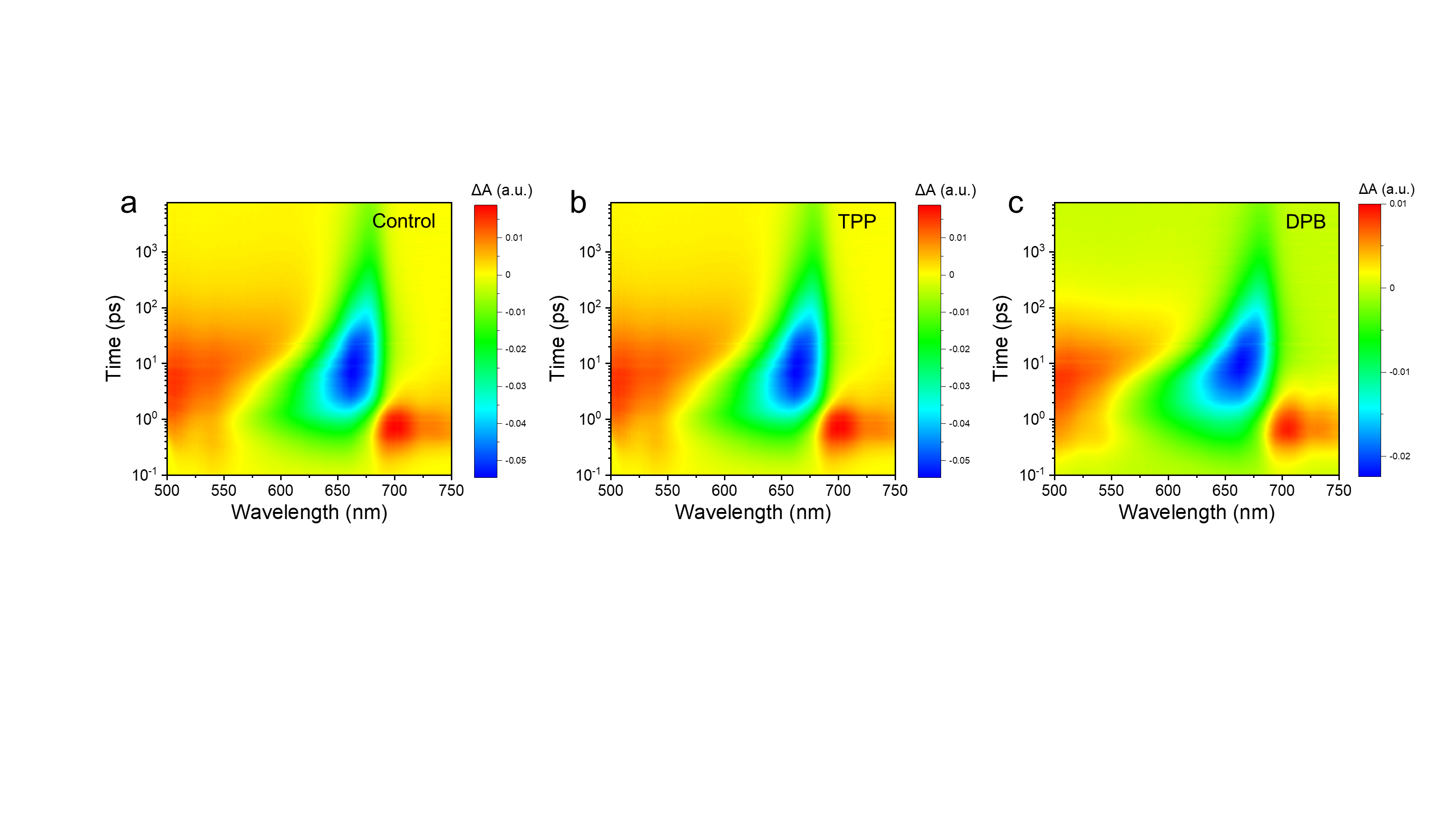


**Fig. S2.** Color-coded contour maps of ΔA for (a) pristine, (b) TPP and (c) DPB passivated CsPbI_3_ NC solution.


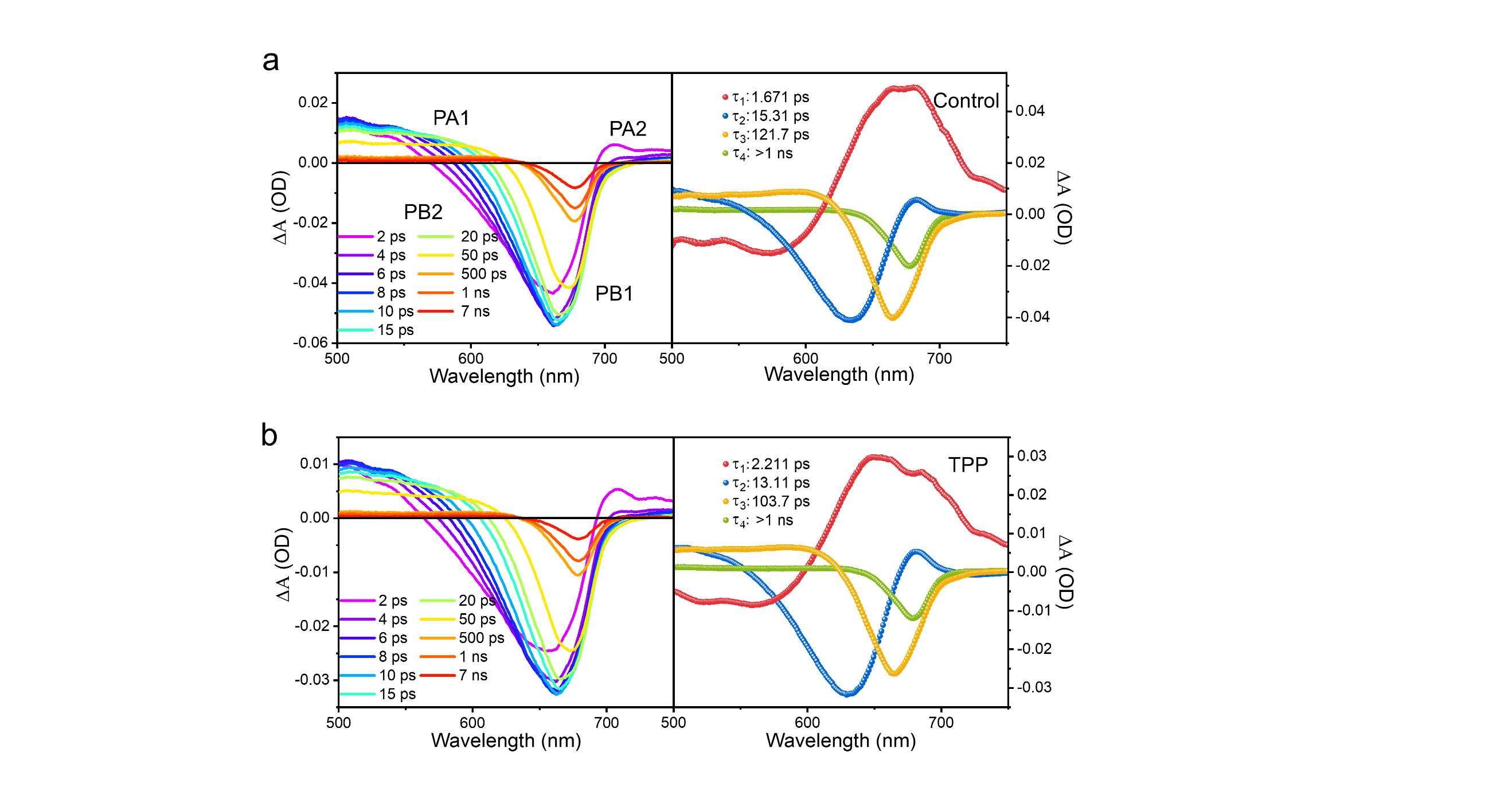


**Fig. S3.** TA spectra taken at several representative probe delays (left panel) and decay-associated spectra (DAS) (right panel) for (a) pristine CsPbI_3_ NCs and (b) TPP passivated CsPbI_3_ NCs.


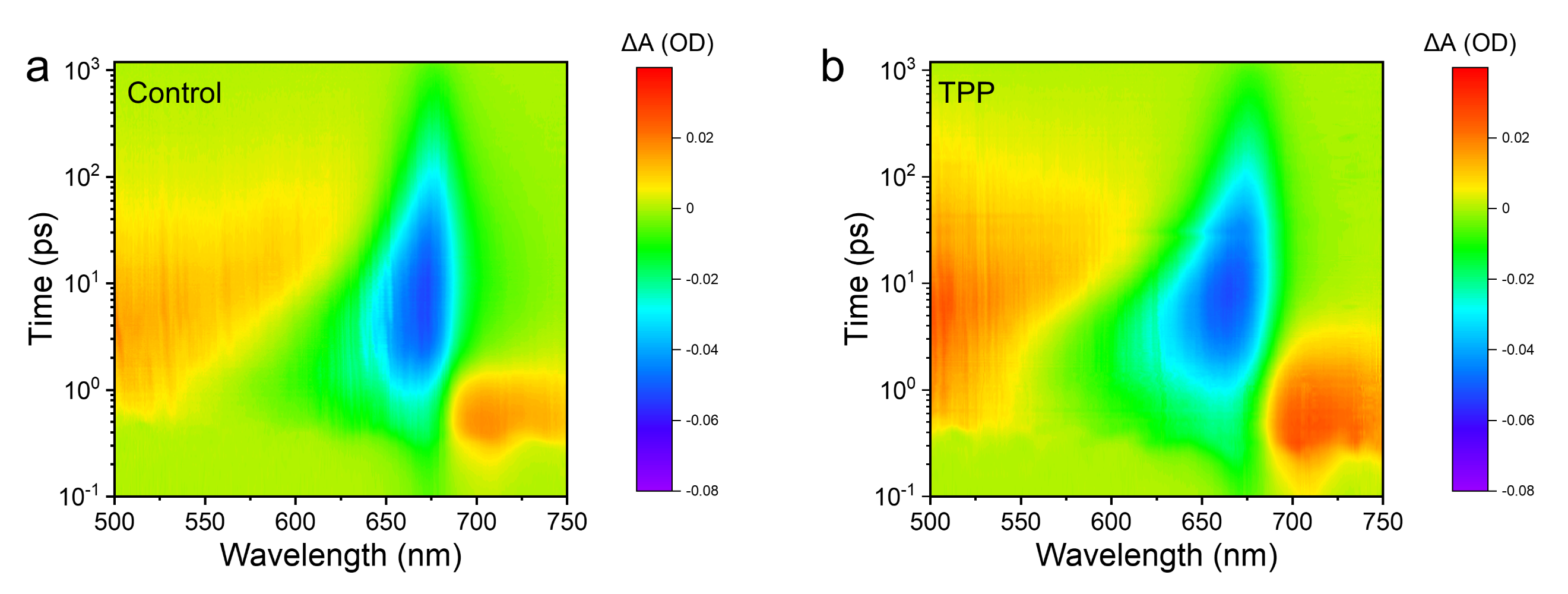


**Fig. S4.** Color-coded contour maps of ΔA for (a) pristine CsPbI_3_ NC films and (b) TPP passivated CsPbI_3_ NC films.


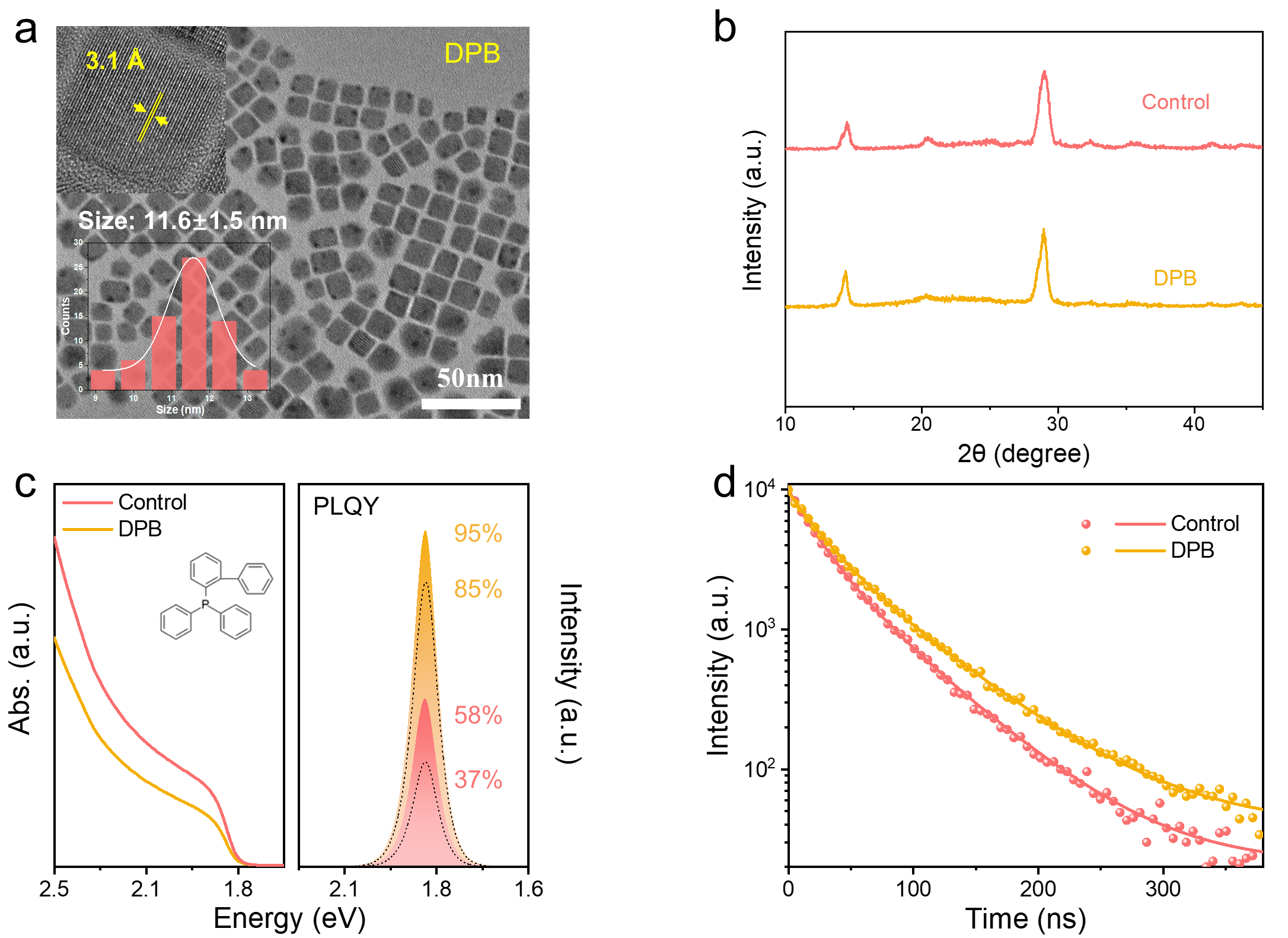


**Fig. S5.** (a) TEM image of NCs passivated with DPB. The top left inset is the high-resolution TEM image and the bottom left inset is the size distribution histograms. (b) XRD patterns of the pristine and DPB passivated NCs. (c) UV−vis absorption spectra (left), PL spectra (right) (The inset is the molecular structural formulas of DPB) and (d) time-resolved PL decay spectra for the pristine and DPB passivated PNCs. (The filled areas are the PLQYs of the solution, and the dot lines are the PLQY of the corresponding films.)


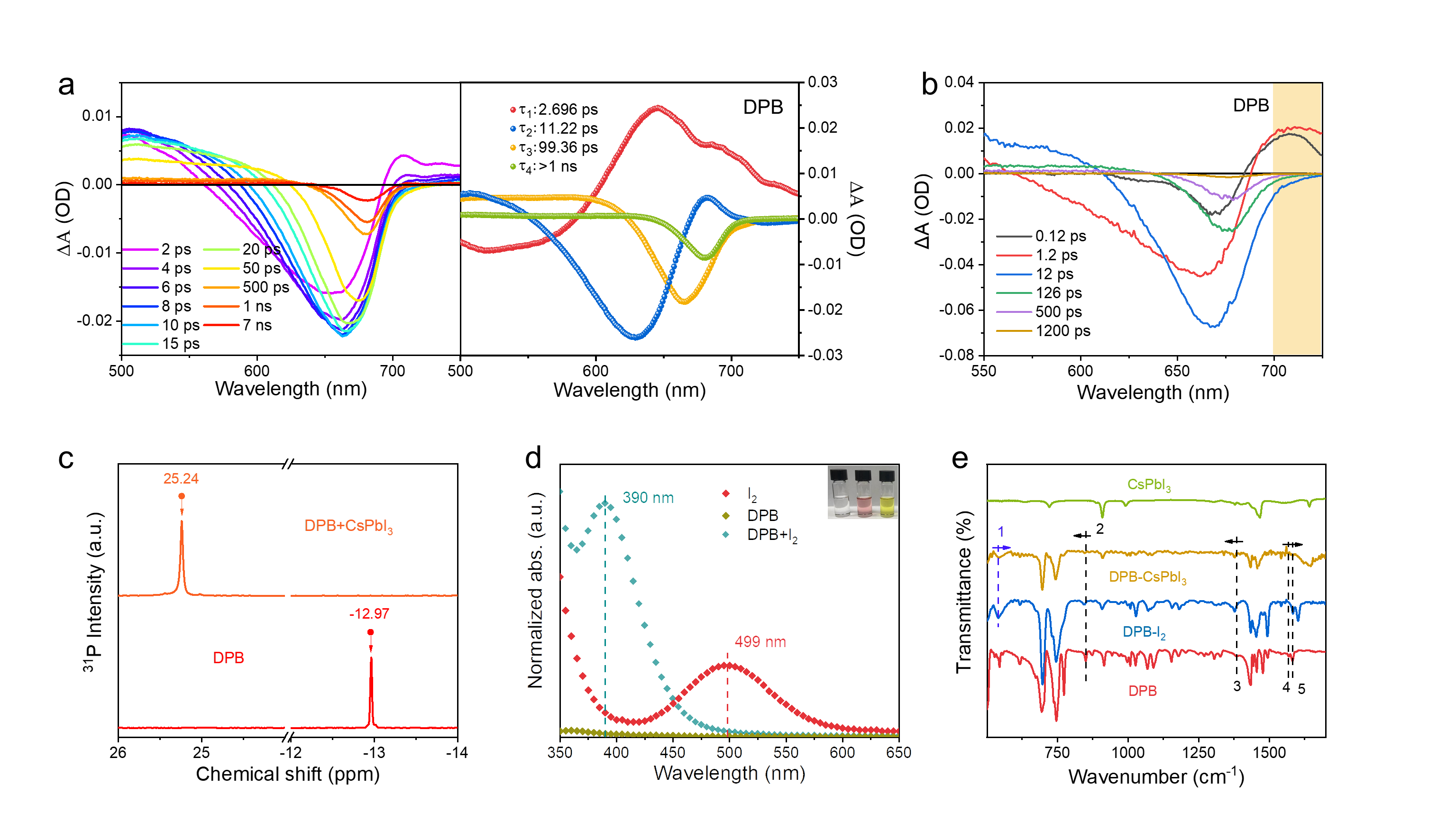


**Fig. S6.** (a) TA spectra taken at several representative probe delays (left panel) and decay-associated spectra (DAS) (right panel) for DPB passivated CsPbI_3_ NC solution. (b) TA spectra of DPB passivated PNC films. The square shaded areas in (b) is to highlight the band tail absorption. (c) ^31^P NMR spectra for DPB and DPB-CsPbI_3_ NCs. (d) UV-vis absorption spectra for I_2_, DPB, and DPB-I_2_. (The inset is the photos of toluene solution including DPB (left), I_2_ (mid) and DPB-I_2_ (right)). (e) FTIR spectra for DPB, DPB-I_2_, DPB-CsPbI_3_ NCs and CsPbI_3_ NCs.


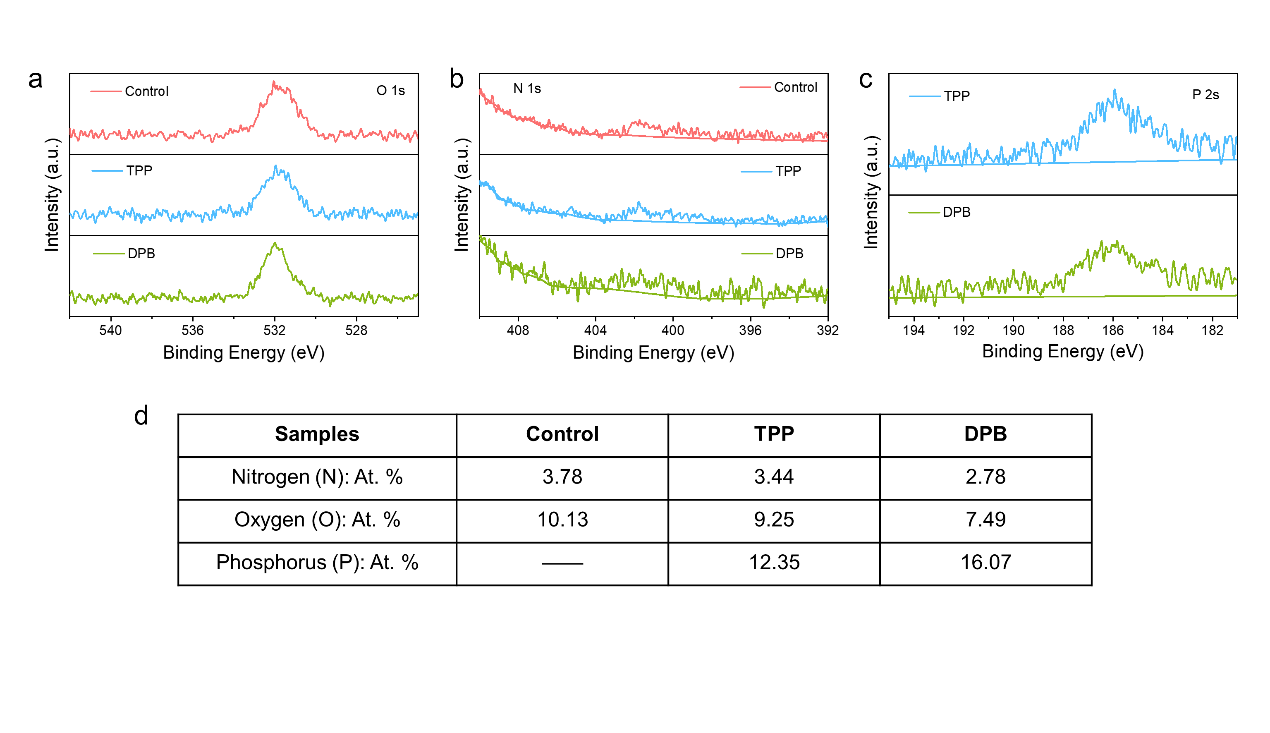


**Fig. S7.** High-resolution XPS spectra of different ligands passivated NCs for (a) O 1s, (b) N 1s and (c) P 2p. (d) The content ratio of different elements.


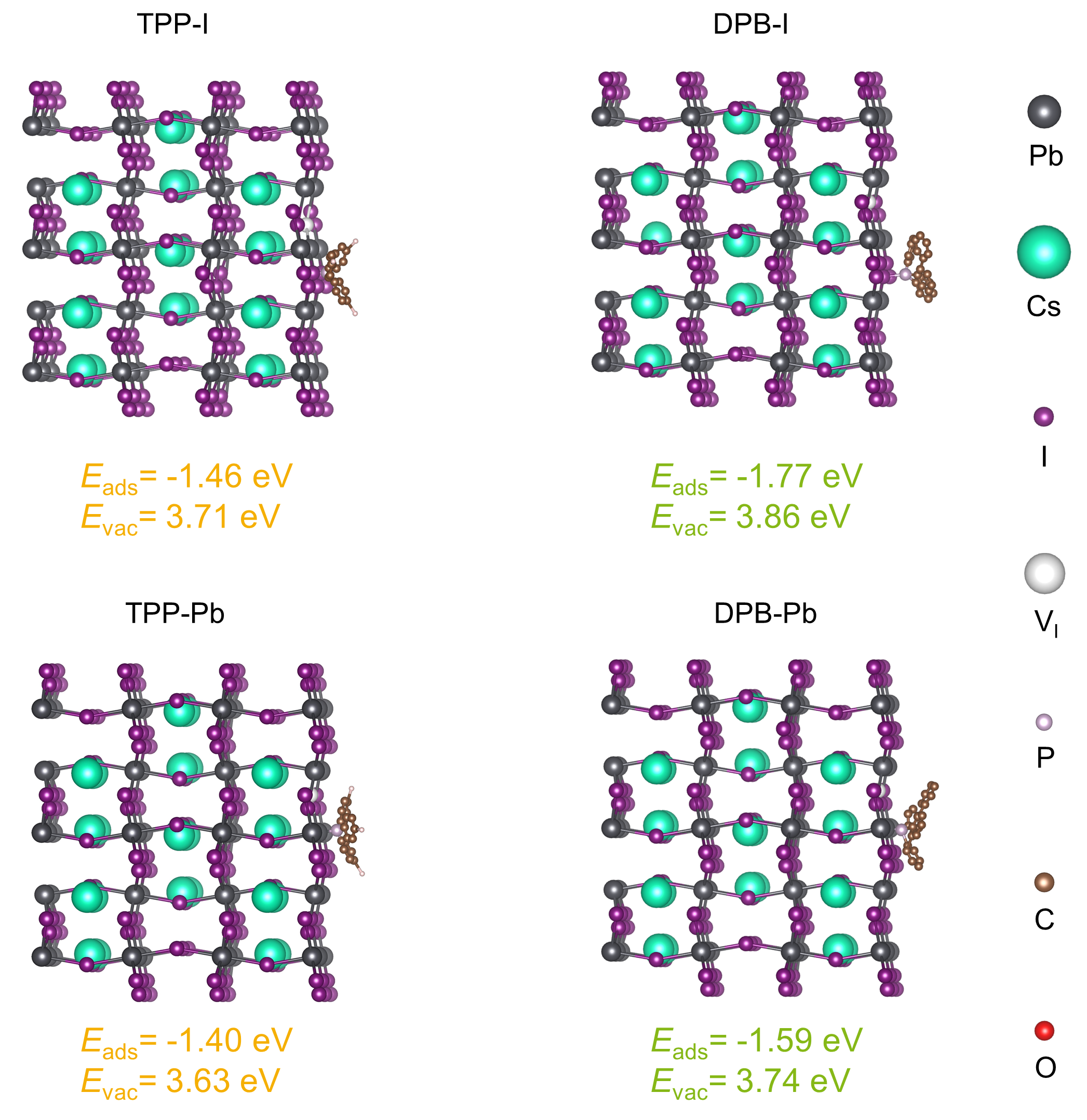


**Fig. S8.** First-principles density functional theory results. Estimated adsorption energy (E_ads_) of different ligands on the CsPbI_3_ PNC surface, and the corresponding formation energy of surface V_I_ (E_vac_).


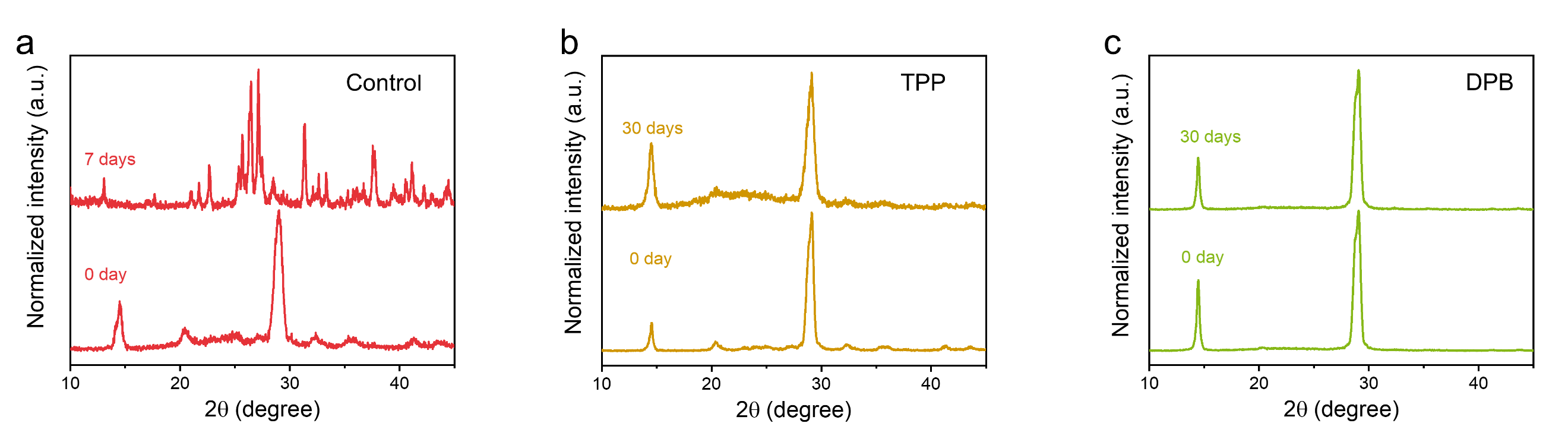


**Fig. S9.** XRD patterns of PNC based on different ligands passivation stored in the air for 30 days.


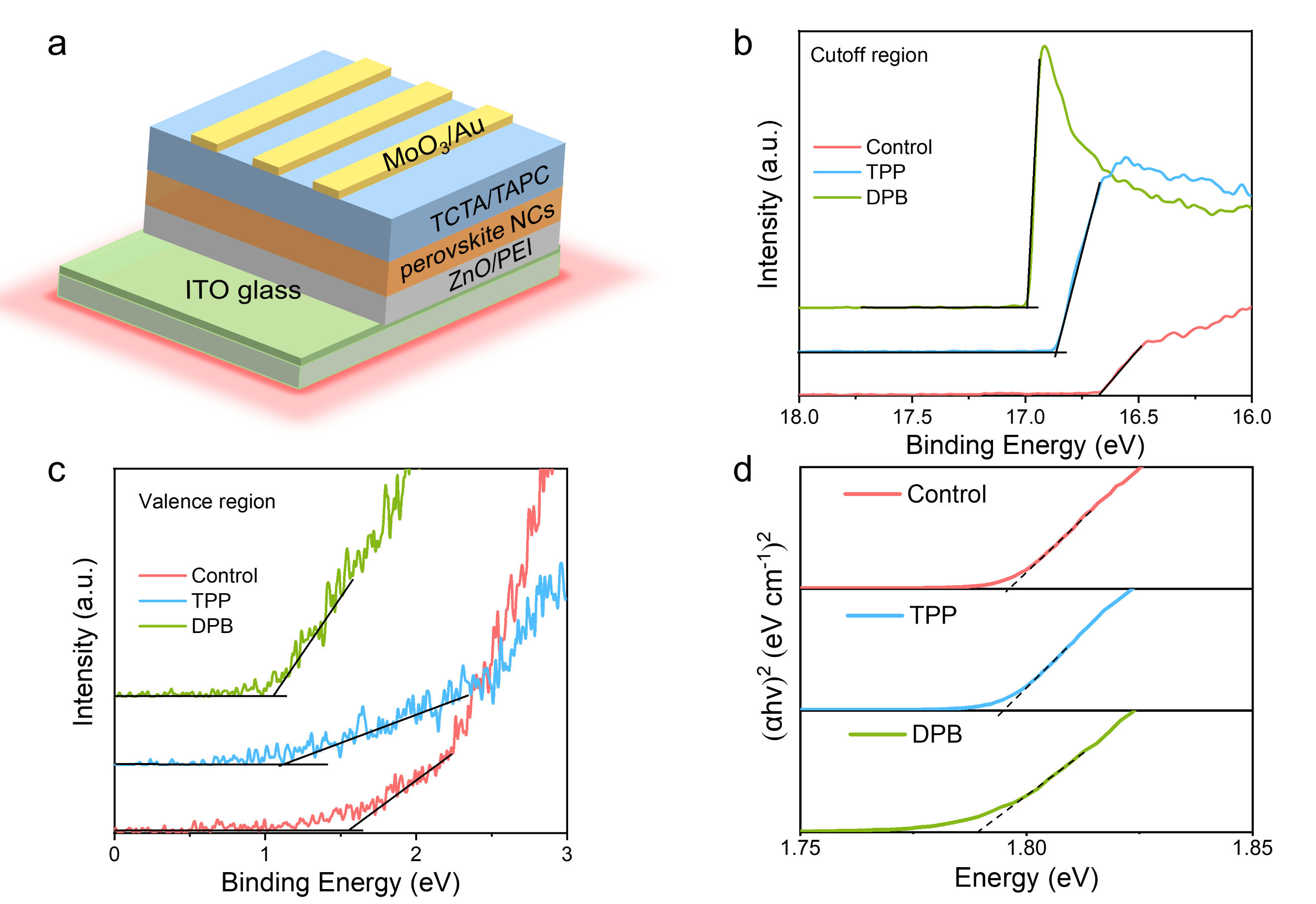


**Fig. S10**. (a) A schematic architecture of PNC based LEDs. (b-c) UPS spectra, (d) Tauc plots of different PNC films deposited on the ITO glass substrate.


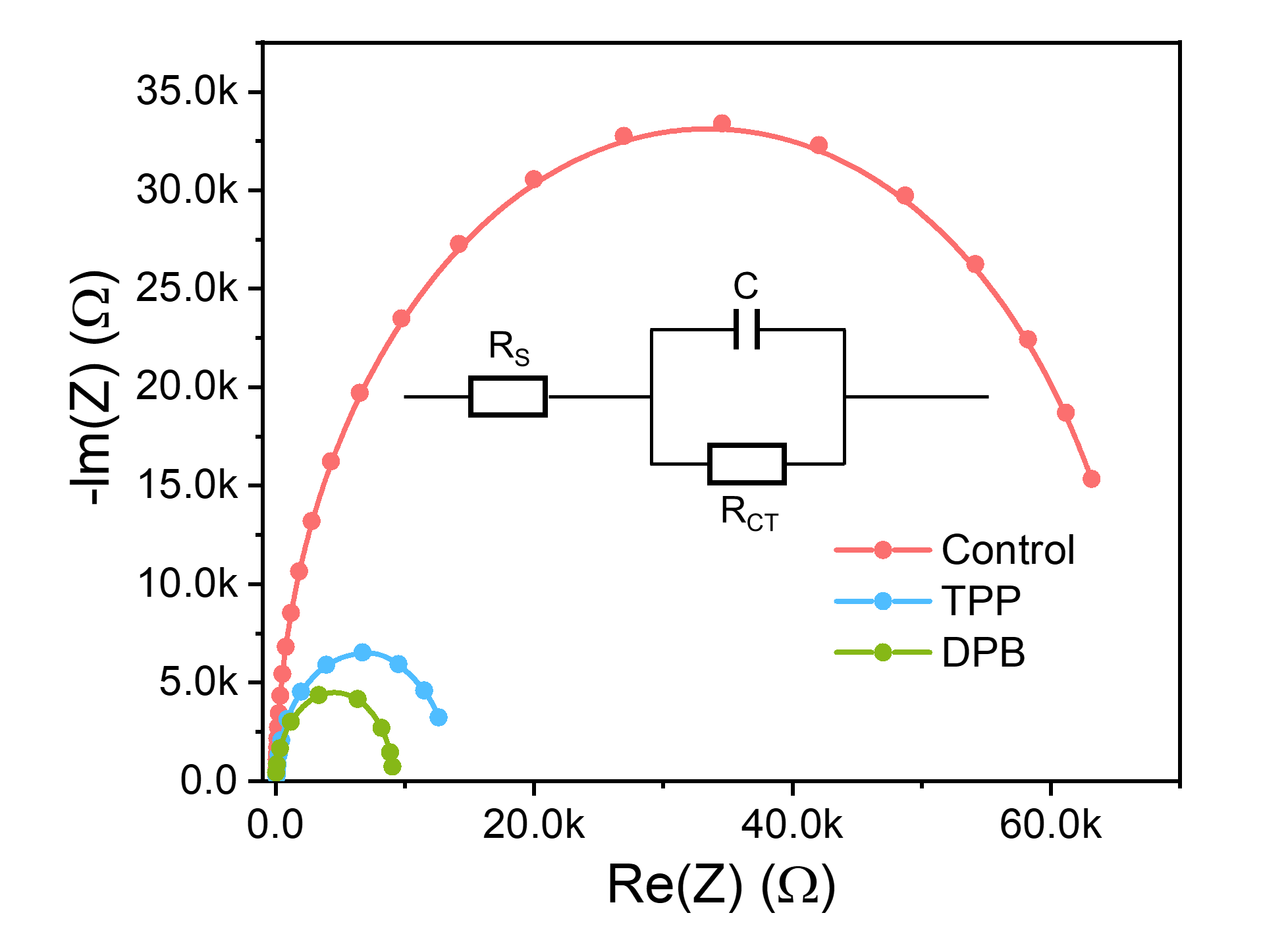


**Fig. S11.** Nyquist spectra of PNC LEDs based on different ligands passivation. The inset is the equivalent circuit of these devices. Note: The passivated devices have smaller series resistance (R_S_) and charge transfer resistance (R_CT_), which implies better charge carrier injection and transfer.^[1](#_ENREF_1)^


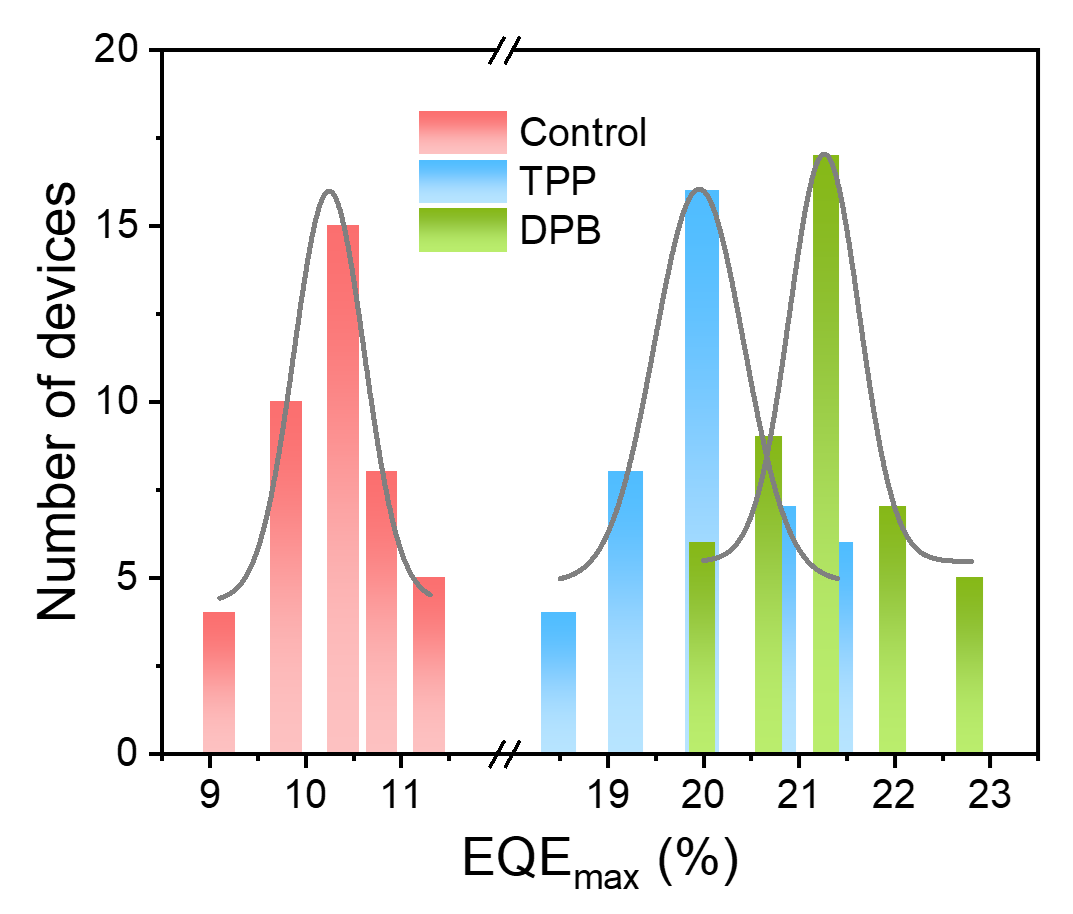


**Fig. S12.** EQE reproducibility of top-emitting PNC LED devices based on different ligand passivation.


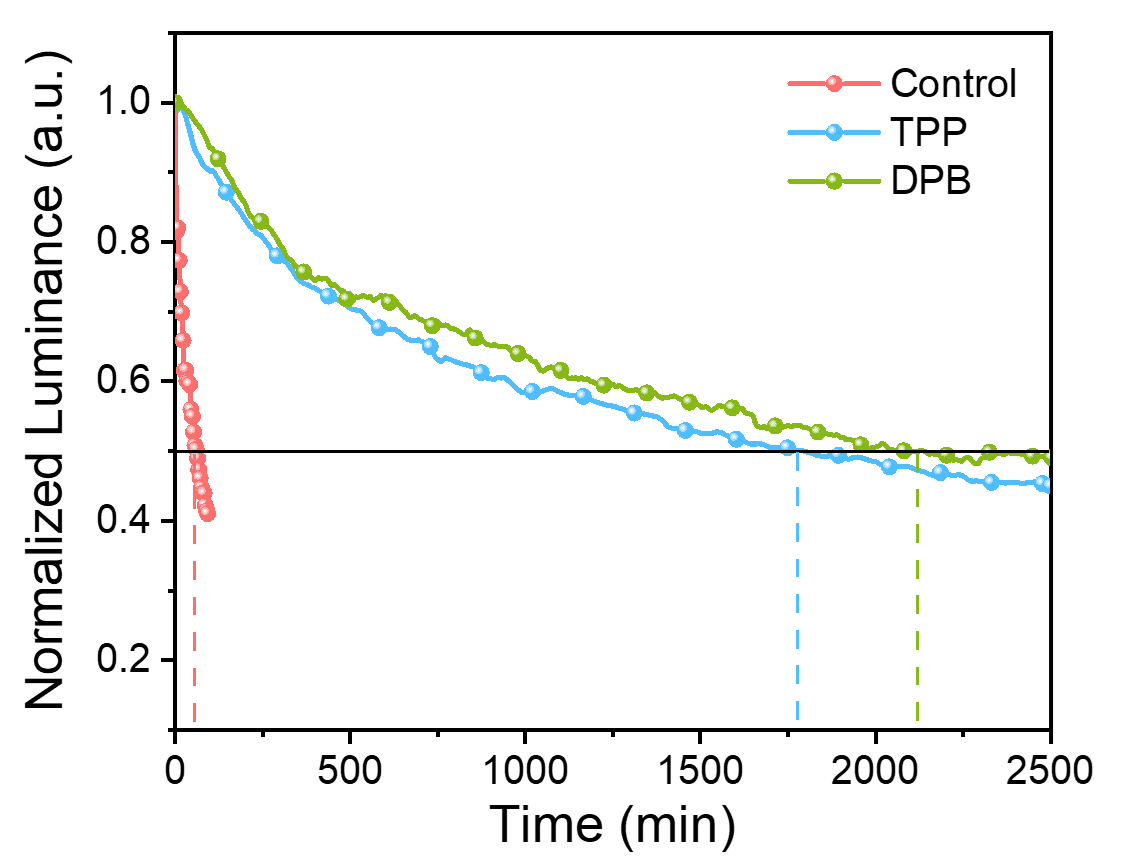


**Fig. S13.** The device lifetimes of different top-emitting LEDs at an initial luminance of 200 cd m^–2^.

**Table S1.** Optical properties including absorption and PL peak positions, PLQYs and average lifetimes of PNCs with different ligands passivation.

| **Sample** | **Abs. peak (nm)** | **PL peak (nm)** | **τ_avg_ (ns)** | **PLQY (%)** |
| --- | --- | --- | --- | --- |
| **Control** | 678 | 688 | 43 | 58 |
| **TPP** | 678 | 688 | 50 | 93 |
| **DPB** | 678 | 688 | 52 | 95 |

**Table S2**. Carrier mobilities of PNC films with different ligands passivation calculated by SCLC method.

| **Sample** | **μ_e_ (cm^2^ V^-1^ s^-1^)** | **μ_h_ (cm^2^ V^-1^ s^-1^)** |
| --- | --- | --- |
| **Control** | 1.3×10^-3^ | 3.0×10^-4^ |
| **TPP** | 2.0×10^-3^ | 7.8×10^-4^ |
| **DPB** | 2.8×10^-3^ | 9.2×10^-4^ |

**Table S3**. Series resistance (R_S_) and charge transport resistance (R_CT_) of PNC LEDs with different ligands passivation.

| **Sample** | **R_S_ (Ω)** | **R_CT_ (Ω)** |
| --- | --- | --- |
| **Control** | 69.8 | 66816 |
| **TPP** | 48.3 | 13168 |
| **DPB** | 44.2 | 9027 |

**Table S4:** EL characteristics, including the efficiency roll-off, of several recently reported blue, green and red emitting perovskite LEDs. (Statistical range (EQE): red >15%, green >20%, blue >10%)

| Perovskite | EL (nm) | Luminance_max_ (cd m^-2^) | EQE_max_ (%) | EQE (%) @ 10 mA cm^-2^ | EQE (%) @100 mA cm^-2^ | EQE (%) @500 mA cm^-2^ | Efficiency roll-off (%) | Reference |
| --- | --- | --- | --- | --- | --- | --- | --- | --- |
| MAPb(I*_x_*Br_1−_*_x_*)_3_ NCs | 620 | 627 | 20.3 | 3.5 |  |  | 82.8 | [^2^](#_ENREF_2) |
| CsPbI_3_ NCs | 640 | ~2000 | 23 | 12 |  |  | 47.8 | [^3^](#_ENREF_3) |
| CsPb(I_1-x_Br_x_)_3_ NCs | 653 | 500 | 21.3 | 7 |  |  | 67.1 | [^4^](#_ENREF_4) |
| CsPbI_3_ QDs | 678 | ~1500 | 20.8 | 10 |  |  | 51.9 | [^5^](#_ENREF_5) |
| CsPbI_3_: Zn NCs | 682 | 2202 | 15.1 |  |  | 5.5 | 63.6 | [^6^](#_ENREF_6) |
| CsPbI_3_: Zn NCs | 687 | 3714 | 15.3 |  |  | 12 | 21.5 | [^1^](#_ENREF_1) |
| CsPbI_3:_ Sr NCs | 687 | 586 | 17.1 |  | 4 |  | 76.6 | [^7^](#_ENREF_7) |
| CsPbI_3_ NCs | 689 | 618 | 17.8 | 12 |  |  | 32.6 | [^8^](#_ENREF_8) |
| CsPbI_3_ NCs | 687 | 576 | 21.8 |  | 3 |  | 86.2 | [^9^](#_ENREF_9) |
| CsPbI_3_ NCs | 688 | 13475 | 21.4 |  |  | 20.4 | 4.7 | This work |
|  | 688 | 15204 | 22.8 |  |  | 22.2 | 2.6 |  |
| FAPbI_3_ 3D | 780 |  | 19.6 |  |  | 15 | 23.5 | [^10^](#_ENREF_10) |
| FAPbI_3_ 3D | 800 |  | 21.6 |  |  | 15.5 | 28.2 | [^11^](#_ENREF_11) |
| FAPbI_3_ 3D | 800 |  | 23.8 |  |  | ~12 | 49.5 | [^12^](#_ENREF_12) |
| FAPbI_3_ 3D | 802 |  | 18.6 |  |  | 8.5 | 54.3 | [^13^](#_ENREF_13) |
| FAPbI_3_ 3D | 802 |  | 17.3 |  |  | 10 | 42.2 | [^14^](#_ENREF_14) |
| FAPbI_3_ 3D | 803 |  | 22.8 |  |  | 15 | 34.2 | [^15^](#_ENREF_15) |
| (BABr)_0.4_ CsPbBr_3_ quasi-2D | 512 | 80000 | 24.5 |  | 8 |  | 67.3 | [^16^](#_ENREF_16) |
| (BABr)_0.4_ CsPbBr_3_ quasi-2D | 512 | 13,400 | 20.5 |  | 5 |  | 75.6 | [^17^](#_ENREF_17) |
| (PEABr)_0.4_ CsPbBr_3_ quasi-2D | 514 | 40514 | 28.1 |  | 9 |  | 68.0 | [^18^](#_ENREF_18) |
| PEA_2_Cs_1.6_MA_0.4_Pb_3_Br_10_ quasi-2D | 517 | 5200 | 25.6 |  | 14 |  | 45.3 | [^19^](#_ENREF_19) |
| p-FPEA_2_MA_n-1_Pb_n_Br_3n+1_ quasi-2D | 530 | 82480 | 20.4 |  | 17.5 |  | 14.2 | [^20^](#_ENREF_20) |
| PEA_2_(FAPbBr_3_)*_2_*PbBr_4_ quasi-2D | 530 | 128842 | 25.3 |  | 20 |  | 20.9 | [^21^](#_ENREF_21) |
| CsPbBr_3_ QDs | 515 | ~30000 | 21.6 |  | 10 |  | 53.7 | [^5^](#_ENREF_5) |
| CsPbBr_3_ NCs | 520 | 41900 | 21.6 |  | 8 |  | 63.0 | [^22^](#_ENREF_22) |
| (FA_0.7_MA_0.1_GA_0.2_)_0.87_Cs_0.13_PbBr_3_ NCs | ~540 | 470000 | 28.9 |  |  | 19 (@ 450 mA cm^-2^) | 34.2 | [^23^](#_ENREF_23) |
| MABr: CsPbBr_3_ 3D | 525 | 14000 | 20.3 | 16 |  |  | 21.2 | [^24^](#_ENREF_24) |
| PEA_2_(Cs_0.4_EA_0.6_PbBr_3_)_2_PbBr_4_  quasi-2D | 488 | 2191 | 12.1 | 7 |  |  | 42.1 | [^25^](#_ENREF_25) |
|  | 495 | 2790 | 13.3 | 10 |  |  | 24.8 |  |
| CsPbBr_3_ QDs | 463  478 | ~600  ~3000 | 10.3  17.9 | 6  10.5 |  |  | 41.7  41.3 | [^5^](#_ENREF_5) |
| CsPbBr_3_: NaBr NCs | 479 | 500 | 12.3 | 3.5 |  |  | 71.5 | [^26^](#_ENREF_26) |
| Rb_0.1_Cs_1.2_FA_0.2_Pb(Br_0.6_Cl_0.4_)_3.5_ 3D | 477 | 2180 | 11 | 8 |  |  | 27.3 | [^27^](#_ENREF_27) |
| CsPbBr_1.5_Cl_1.5_ 3D | 486 | 1390 | 12.8 | 7 |  |  | 45.3 | [^28^](#_ENREF_28) |

# References

1. Shen, X. Y*. et al.* Silver–Bismuth Bilayer Anode for Perovskite Nanocrystal Light-Emitting Devices. *Journal of Physical Chemistry Letters* **11**, 3853-3859 (2020).

2. Hassan, Y. *et al.* Ligand-engineered bandgap stability in mixed-halide perovskite LEDs. *Nature* **591**, 72-77 (2021).

3. Wang, Y. K. *et al.* All-Inorganic Quantum-Dot LEDs Based on a Phase-Stabilized α-CsPbI_3_ Perovskite. *Angewandte Chemie International Edition* **60**, 16164-16170 (2021).

4. Chiba, T. *et al.* Anion-exchange red perovskite quantum dots with ammonium iodine salts for highly efficient light-emitting devices. *Nature Photonics* **12**, 681-687 (2018).

5. Jiang Y. *et al.* Synthesis-on-substrate of quantum dot solids. *Nature* **612**, 679-684 (2022).

6. Shen, X. Y. *et al.* Zn-Alloyed CsPbI_3_ Nanocrystals for Highly Efficient Perovskite Light-Emitting Devices. *Nano Letts* **19**, 1552-1559 (2019).

7. Chen C. *et al.* Highly stable CsPbI_3_:Sr^2+^ nanocrystals with near-unity quantum yield enabling perovskite light-emitting diodes with an external quantum efficiency of 17.1%. *Nano Energy* **85**, 106033 (2021).

8. Li, H. M. *et al.* Efficient and Stable Red Perovskite Light-Emitting Diodes with Operational Stability >300 h. *Advanced Materials* **33**, 2008820 (2021).

9. Chen C. *et al.* Passivation Layer of Potassium Iodide Yielding High Efficiency and Stable Deep Red Perovskite Light-Emitting Diodes. *ACS Applied Materials & Interfaces* **14**, 16404-16412 (2022).

10. Yuan Z. *et al.* Unveiling the synergistic effect of precursor stoichiometry and interfacial reactions for perovskite light-emitting diodes. *Nature Communications* **10**, 2818 (2019).

11. Xu W. *et al.* Rational molecular passivation for high-performance perovskite light-emitting diodes. *Nature Photonics* **13**, 418-424 (2019).

12. Sun Y. *et al.* Bright and stable perovskite light-emitting diodes in the near-infrared range. *Nature* **615**, 830-835 (2023).

13. Kuang C. *et al.* Critical role of additive-induced molecular interaction on the operational stability of perovskite light-emitting diodes. *Joule* **5**, 618-630 (2021).

14. Wang H. *et al.* Perovskite-molecule composite thin films for efficient and stable light-emitting diodes. *Nature Communications* **11**, 891 (2020).

15. Guo B. *et al.* Ultrastable near-infrared perovskite light-emitting diodes. *Nature Photonics* **16**, 637-643 (2022).

16. Dong W. *et al.* Amine-Terminated Carbon Dots Linking Hole Transport Layer and Vertically Oriented Quasi-2D Perovskites through Hydrogen Bonds Enable Efficient LEDs. *ACS Nano* **16**, 9679-9690 (2022).

17. Kong L. *et al.* Smoothing the energy transfer pathway in quasi-2D perovskite films using methanesulfonate leads to highly efficient light-emitting devices. *Nature Communications* **12**, 1246 (2021).

18. Liu Z. *et al.* Perovskite Light-Emitting Diodes with EQE Exceeding 28% through a Synergetic Dual-Additive Strategy for Defect Passivation and Nanostructure Regulation. *Advanced Materials* **33**, 2103268 (2021).

19. Ma D. *et al.* Distribution control enables efficient reduced-dimensional perovskite LEDs. *Nature* **599**, 594-598 (2021).

20. Jiang Y. *et al.* Reducing the impact of Auger recombination in quasi-2D perovskite light-emitting diodes. *Nature Communications* **12**, 336 (2021).

21. Gao Y. *et al.* High-Performance Perovskite Light-Emitting Diodes Enabled by Passivating Defect and Constructing Dual Energy-Transfer Pathway through Functional Perovskite Nanocrystals. *Advanced Materials* **34**, 2207445 (2022).

22. Fang T. *et al.* Perovskite QLED with an external quantum efficiency of over 21% by modulating electronic transport. *Science Bulletin* **66**, 36-43 (2021).

23. Kim, J. S. *et al.* Ultra-bright, efficient and stable perovskite light-emitting diodes. *Nature* **611**, 688-694 (2022).

24. Lin K. *et al.* Perovskite light-emitting diodes with external quantum efficiency exceeding 20 per cent. *Nature* **562**, 245-248 (2018).

25. Chu Z. *et al.* Large cation ethylammonium incorporated perovskite for efficient and spectra stable blue light-emitting diodes. *Nature Communications* **11**, 4165 (2020).

26. Dong Y. *et al.* Bipolar-shell resurfacing for blue LEDs based on strongly confined perovskite quantum dots. *Nature Nanotechnology* **15**, 668-674 (2020).

27. Karlsson M. *et al.* Mixed halide perovskites for spectrally stable and high-efficiency blue light-emitting diodes. *Nature Communications* **12**, 361 (2021).

28. Shen Y. *et al.* Interfacial Nucleation Seeding for Electroluminescent Manipulation in Blue Perovskite Light-Emitting Diodes. *Advanced Functional Materials* **31**, 2103870 (2021).
